# Association between the *MVK* and *MMAB* polymorphisms and serum lipid levels

**DOI:** 10.18632/oncotarget.19707

**Published:** 2017-07-31

**Authors:** Liu Miao, Rui-Xing Yin, Shang-Ling Pan, Shuo Yang, De-Zhai Yang, Wei-Xiong Lin

**Affiliations:** ^1^ Department of Cardiology, Institute of Cardiovascular Diseases, The First Affiliated Hospital, Guangxi Medical University, Nanning 530021, Guangxi, People’s Republic of China; ^2^ Department of Pathophysiology, School of Premedical Science, Guangxi Medical University, Nanning 530021, Guangxi, People’s Republic of China; ^3^ Department of Molecular Genetics, Medical Scientific Research Center, Guangxi Medical University, Nanning 530021, Guangxi, People’s Republic of China

**Keywords:** mevalonate kinase and methylmalonic aciduria (cobalamin deficiency) cblB type gene, single nucleotide polymorphism, haplotype, lipids, environmental factor

## Abstract

Maonan ethnic group is a relatively conservative and isolated minority in China. Little is known about the association of the mevalonate kinase (*MVK*), methylmalonic aciduria (cobalamin deficiency) cblB type (*MMAB*) single nucleotide polymorphisms (SNPs) and serum lipid levels. This study aimed to determine the association between four SNPs in the *MVK/MMAB* and serum lipid levels. Genotyping of the rs3759387, rs877710, rs7134594 and rs9593 SNPs was performed in 1264 Maonan subjects and 1251 Han participants. Allele and genotype frequencies of the selected SNPs were different between the two populations (*P <* 0.05-0.001). Four SNPs were associated with high-density lipoprotein cholesterol (HDL-C) in the both ethnic groups (*P <* 0.0125-0.001); and one SNP with apolipoprotein (Apo) A1 (rs7134594) in Han Chinese (*P <*0.0125). Strong linkage disequilibria were noted among the SNPs (*D*'=0.63-0.96; *r*^*2*^=0.13-0.88). The commonest haplotype was C-C-C-T (> 50%). The frequencies of C-C-C-T, C-G-T-A, A-G-T-A, C-G-C-T, and A-C-T-A were different between the two populations (*P <*0.001). The associations between haplotypes and dyslipidemia were different in the Han and/or Maonan population (*P <* 0.05-0.001), haplotypes could explain much more serum lipid variation than any single SNP alone especially for HDL-C. Differences in lipid profiles between the two populations might partially attribute to these SNPs and their haplotypes.

## INTRODUCTION

With the remarkable improvement of social living standard, coronary artery disease (CAD) and ischemic stroke, as the major reason, contribute to increasing morbidity, mortality and disability in developing countries. Maybe the rates would be keeping on increasing [1]. Recently, lots of epidemiological researches have demonstrated an inverse effect between serum high-density lipoprotein cholesterol (HDL-C) levels and CAD morbidity and mortality [2–4]. In addition, each 1% decrease in low-density lipoprotein cholesterol (LDL-C) levels can give rise to 1% decrease the risk of CAD [5], but each 2% increase the risk of CAD can result from each 1% HDL-C levels decrease [6]. Previous researches about cardiovascular disease risk factors which have well-known for us that they would be raised by different gender [7], also be distinguish from age [8] and ethnicity [9], and would be modified by behavioral choices [10], poor diet [11] and exercise lifestyle [12], environmental factors [13], and personal genetic profile [14, 15]. As regards this point, genetic factors can determine approximate 50% of the variation in HDL-C levels according to family and twin researches [16, 17].

Recent GWASes have found several novel loci located at chromosome 12q24, which covers the mevalonate kinase gene (*MVK*, also known as: *MK; LRBP; MVLK; POROK3*, Gene ID: 4598, HGNC ID: 7530), methylmalonic aciduria (cobalamin deficiency) cblB type gene (*MMAB*, also known as: *ATR; cob; cblB; CFAP23*, Gene ID: 362265, HGNC ID: 19331), all of which can affect HDL-C levels [18, 19]. Because of having effect on lipid levels, this important region has also been revealed by other linkage researches [20–22]. Particularly, *MVK* and *MMAB,* as two adjacent genes, take part in metabolic pathways, would also be influenced HDL-C metabolism [23]. A previous GWAS on plasma lipid levels has identified the rs877710 SNP near the *MMAB* as hyperlipidemia loci in Mexicans and have demonstrated in might be ethnic-specificity [24]. A study in 2011 has shown that the association between the *MMAB* rs7134594 SNP and *MVK* rs3759387 SNP and serum lipid levels might have sex-specificity [25]. Sun *et al* had taken several SNPs into consideration that including *MVK* (rs3759387, rs2287218) and *MMAB* (rs12817689, rs22411201, rs11067227, rs7134594, rs877710, rs11067233, rs9593, rs11831226, rs8228), but only found that rs11067233 in *MMAB* may contribute to the susceptibility of CAD by altering plasma HDL-C levels in Han Chinese [26]. Junyent *et al* suggested that *MMAB*-3U3527G/C variants might result in the variation in HDL-C levels, particularly in those individuals with high carbohydrate consumptions [27]. Fogarty *et al* had found that *MMAB* rs9593 might be have some effect on serum HDL-C, but they were not sure of that [28]. Whether the *MVK/MMAB* SNPs are associated with serum lipid levels or whether it shows ethnic- and/or sex-specific association, as the previously reported or not, it remains to explore.

As a multi-ethnic country, China contains 56 ethnic groups. Among all of these ethnic groups, Han is the largest one, and Maonan is one of the 55 minorities with a population of 107,166 (Rank 37) according to the sixth national census statistics of China in 2010. Several previous studies have showed that the genetic relationship between Maonan ethnic group and other ethnic groups in Guangxi [29] was much closer than that between Maonan and Han ethnic groups [30]. The special customs and culture, including their clothing, intra-ethnic marriages, dietary habits and lifestyle factors are different from those of local Han Chinese [31]. The previous studies had shown that sexual dimorphism has been demonstrated as the potential of dyslipidemia. The current study, therefore, was undertaken to find out the association of *MVK* rs3759387 and *MMAB* rs7134594, rs877710, rs9593 SNPs and several environmental factors with serum lipid concentrations between sex differences in the Maonan and Han populations.

## RESULTS

### General characteristics of the subjects

The general characteristics of the subjects between two ethnic groups are shown in Table 1. The values of body weight, body mass index (BMI), waist circumference and the percentages of individuals who alcohol consumed, systolic blood pressure, diastolic blood pressure, pulse pressure and blood glucose level were higher in Maonan than in Han (*P* < 0.05-0.001), whereas the values of body height, smoked cigarettes percent, HDL-C and apolipoprotein (Apo) A1 levels were lower in Maonan than in Han (*P* < 0.05). There was no significant difference in the levels of age, sex ratio, total cholesterol (TC), triglyceride (TG), LDL-C, ApoB, and the ratio of ApoA1 to ApoB (*P* > 0.05 for all).

**Table 1:** Comparison of demographic, lifestyle characteristics and serum lipid levels between the Han and Maonan populations

Parameter	Han	Maonan	*Test-statistic*	*P*
Number	1251	1264		
Male/female	476/775	517/747	2.024	0.155
Age (years)^1^	55.88±13.89	56.97±15.12	1.682	0.195
Height (cm)	153.93±7.48	153.81±8.17	8.175	0.004
Weight (kg)	52.69±8.69	53.43±10.75	41.013	1.6×10^-9^
Body mass index (kg/m^2^)	22.23±3.26	22.42±3.70	5.014	0.025
Waist circumference	74.91±7.78	76.74±9.07	23.110	2.2×10^-9^
Smoking status [*n* (%)]				
Non-smoker	936(74.80)	990(78.32)		
≤ 20 cigarettes/day	275(22.10)	244(19.30)		
> 20 cigarettes/day	40(3.10)	30(2.38)	4.727	0.094
Alcohol consumption [*n* (%)]				
Non-drinker	1008(80.58)	997(78.88)		
≤ 25 g/day	106(8.47)	152(12.03)		
> 25 g/day	137 (10.95)	115(9.10)	10.1162	0.006
Systolic blood pressure (mmHg)	128.44±19.72	136.12±23.28	29.984	8×10^-7^
Diastolic blood pressure (mmHg)	80.65±10.83	83.32±11.88	9.067	0.003
Pulse pressure (mmHg)	47.78±14.72	52.80±17.41	29.277	7×10^-7^
Glucose (mmol/L)	6.16±1.78	6.20±1.42	24.878	5.5×10^-7^
Total cholesterol (mmol/L)	4.95±1.06	5.00±1.25	0.392	0.532
Triglyceride (mmol/L)^2^	1.39(0.69)	1.43(0.71)	0.037	0.854
HDL-C (mmol/L)	1.97±0.85	1.67±1.25	6.828	0.021
LDL-C (mmol/L)	2.87±0.84	2.88±0.79	1.606	0.205
ApoA1 (g/L)	1.40±0.33	1.34±0.40	4.883	0.043
ApoB (g/L)	0.84±0.20	0.88±0.20	2.898	0.089
ApoA1/ApoB	1.75±0.58	1.60±0.64	1.632	0.202

*HDL-C*, high-density lipoprotein cholesterol; *LDL-C*, low-density lipoprotein cholesterol; Apo, apolipoprotein. The value of triglyceride was presented as median (interquartile range) for not a normal distribution, the difference between the two ethnic groups was determined by the Wilcoxon-Mann-Whitney test.

^1^ Mean ± SD determined by *t*-test.

^2^ Median (interquartile range) tested by the *Wilcoxon-Mann-Whitney* test.

### Genotypic and allelic frequencies

**Figure 1:**
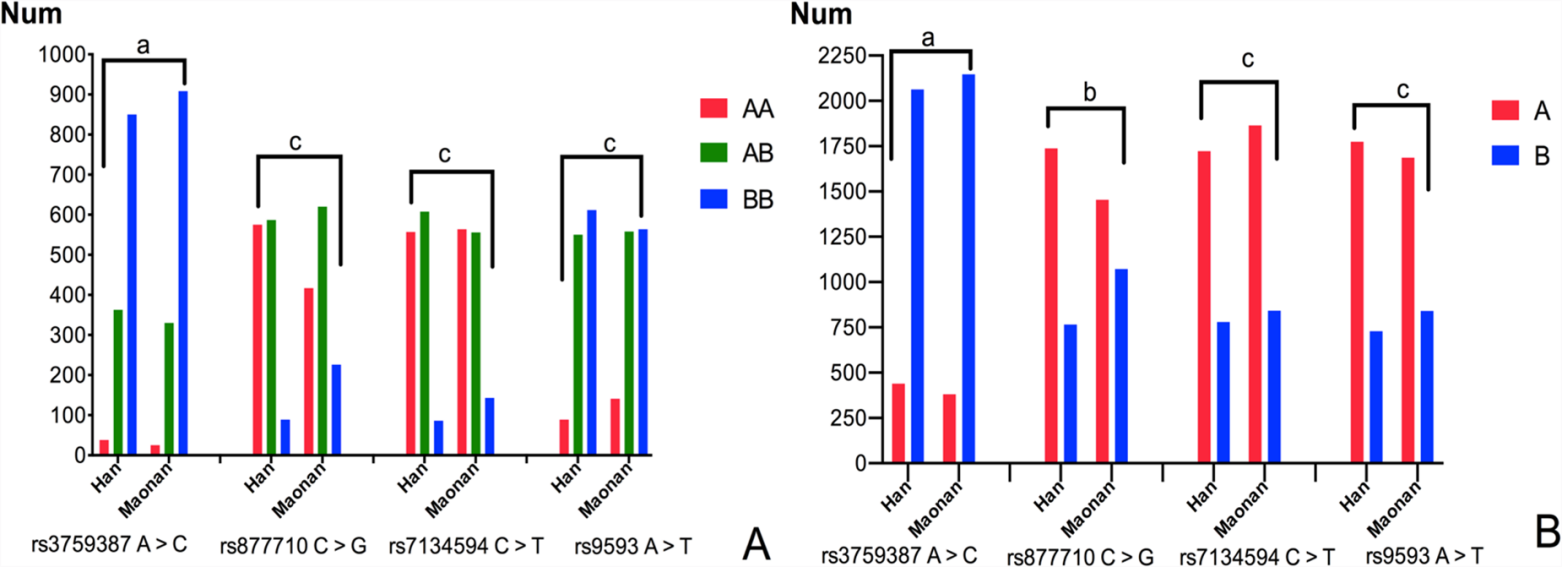
Frequencies of the genotype (A) and allele (B) in the Han and Maonan populations (A) *AA*, rs3759387AA, rs877710CC, rs7134594CC, and rs9593AA genotypes; *AB*, rs3759387AC, rs877710CG, rs7134594CT, and rs9593AT genotypes; *BB*, rs3759387CC, rs877710GG, rs7134594TT, and rs9593TT genotypes. (B) *A*, rs3759387A, rs877710C, rs7134594C, and rs9593A alleles; *B*, rs3759387C, rs877710G, rs7134594T, and rs9593T alleles; ^a^*P* < 0.05; ^b^*P* < 0.01; ^c^*P* < 0.001; *P*_HWE_ > 0.05 for all.

Figure 1 describes the allelic and genotypic frequencies of the selected SNPs. The frequencies of genotype were performed as AA (3.04% *vs*. 1.98%), AC (29.02% *vs*. 26.13%) and CC (67.95% *vs*. 71.89%) in rs3759387 when compared with Han and Maonan minority. The same situations in rs877710 were CC (45.96% *vs*. 33.01%), CG (46.92% *vs*. 49.09%) and GG (7.11% *vs*. 17.89%), rs7134594 were CC (44.52% *vs*. 44.65%), CT (48.60% *vs*. 44.02%) and TT (6.87% *vs*. 11.32%), rs9593 were AA (7.11% *vs*. 11.16%), AT (43.96% *vs*. 44.18%) and TT (48.92% *vs*. 44.66%), respectively. The frequencies of allele were performed as A (17.55% *vs*. 15.04%) and C (82.45% *vs*. 84.96%) in rs3759387 when compared with Han and Maonan minority. The same situations in rs877710 were C (69.42% *vs*. 61.16%) and G (30.58% *vs*. 38.84%), rs7134594 were C (68.82% *vs*. 73.79%) and T (31.18% *vs*. 26.21%), rs9593 were A (70.90% *vs*. 66.75%) and T (29.10% *vs*. 33.25%), respectively. There were distinguished Han from Maonan ethnic group (*P* < 0.05 for all). Besides, four of the selected SNPs were complied with the Hardy-Weinberg equilibrium (*P* > 0.05).

### Genotypes and plasm lipid concentrations

**Figure 2:**
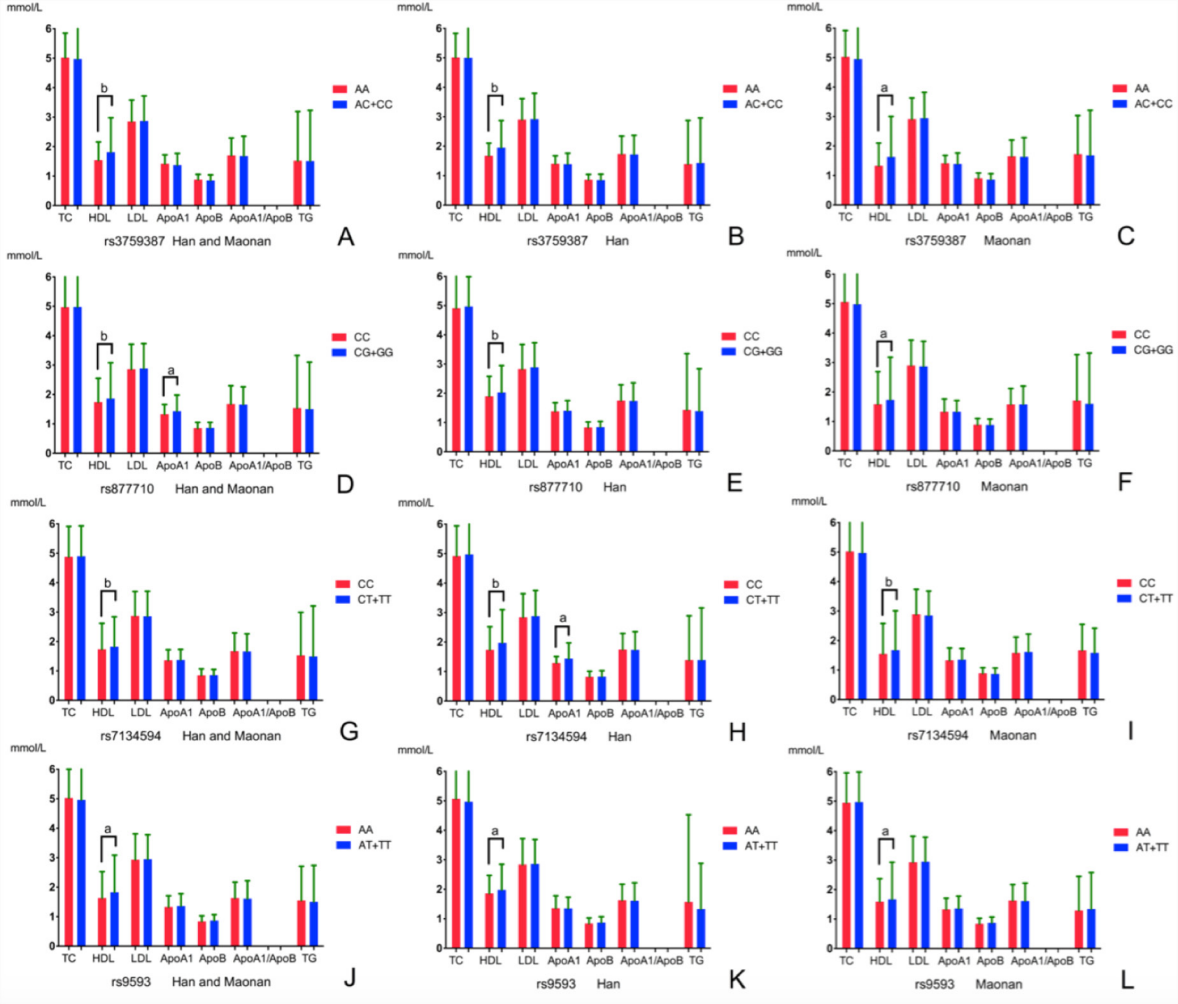
Comparison of the genotypes and serum lipid levels between the Han and Maonan populations *TC,* total cholesterol; *TG*, triglyceride; *HDL-C*, high-density lipoprotein cholesterol; *LDL-C*, low-density lipoprotein cholesterol; *ApoA1*, apolipoprotein A1; *ApoB*, apolipoprotein B; *ApoA1/ApoB*, the ratio of apolipoprotein A1 to apolipoprotein B. The value of triglyceride was presented as median (interquartile range), the difference among the genotypes was determined by the Kruskal-Wallis test. *P* < 0.0125 (corresponding to *P* < 0.05 after adjusting for 4 independent tests by the Bonferroni correction) was considered statistically significant. The minor allele carriers had higher serum HDL-C and/or ApoA1 levels than the minor allele non-carriers. ^a^*P* < 0.0125; ^b^*P* < 0.001.

Figure 2 shows the association between genotypes and plasm lipid concentrations. When we analyzed the dominant model associated with serum lipid parameters we can find that the minor allele carriers had higher HDL-C concentrations than the minor allele non-carriers in Han and/or Maonan ethic groups (*P* < 0.0125 for each). Also, we had found that the minor allele carriers had higher ApoA1 concentrations than the minor allele non-carriers in the combined population of Han and Maonan (rs877710) or only in Han ethic group (rs7134594) (*P* < 0.0125 for each).

### Haplotypes and serum lipid levels

**Figure 3:**
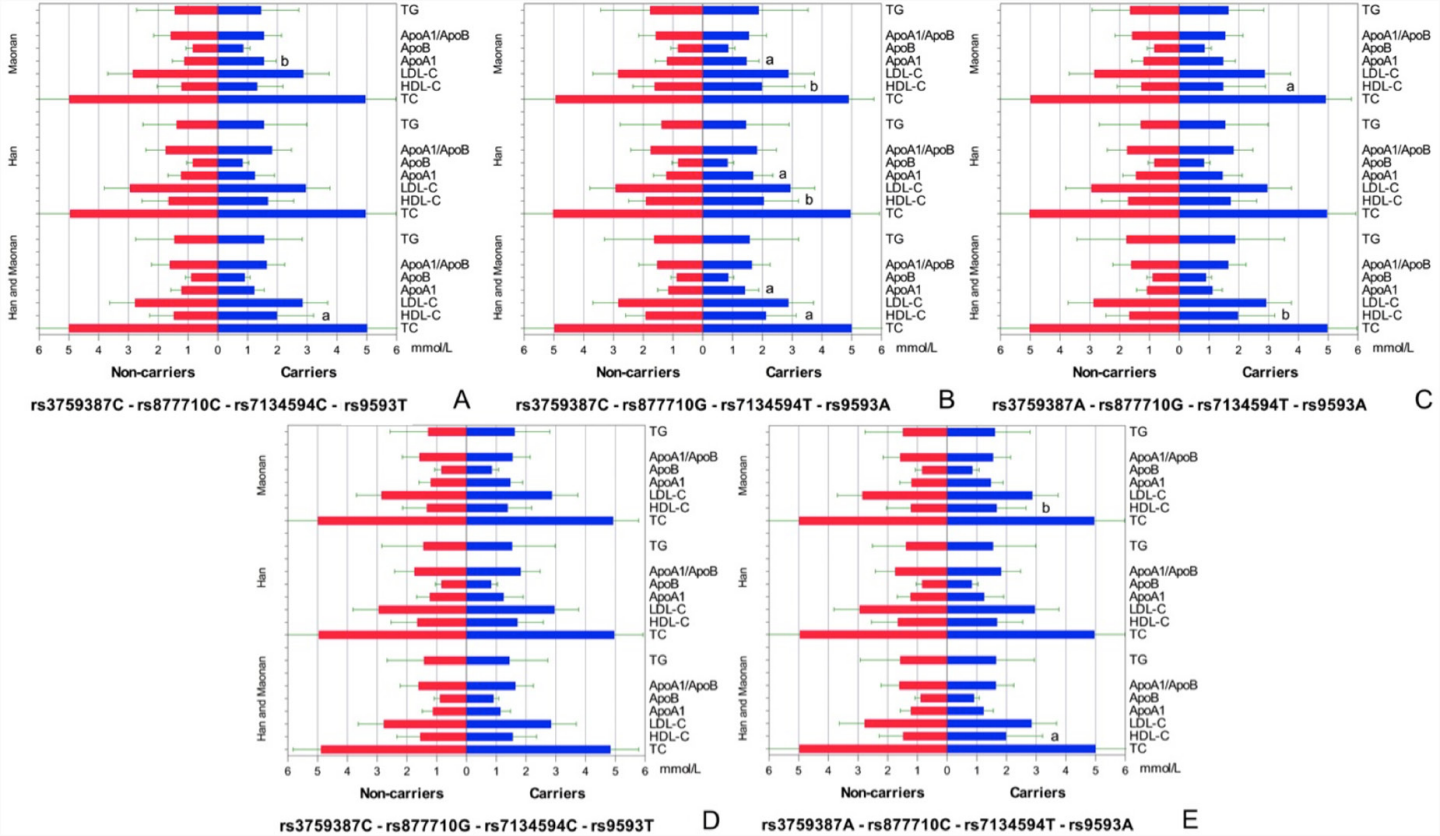
Lipid profiles according to the haplotypes of the two ethnic groups *P* < 0.0125 (corresponding to *P* < 0.05 after adjusting for 4 independent tests by the Bonferroni correction) was considered statistically significant. All of the detected haplotype carriers had higher serum HDL-C and/or ApoA1 levels than the haplotype non-carriers except for rs3759387C-rs877710G-rs7134594C-rs9593T carriers. ^a^*P* < 0.0125; ^b^*P* < 0.001.

**Figure 4:**
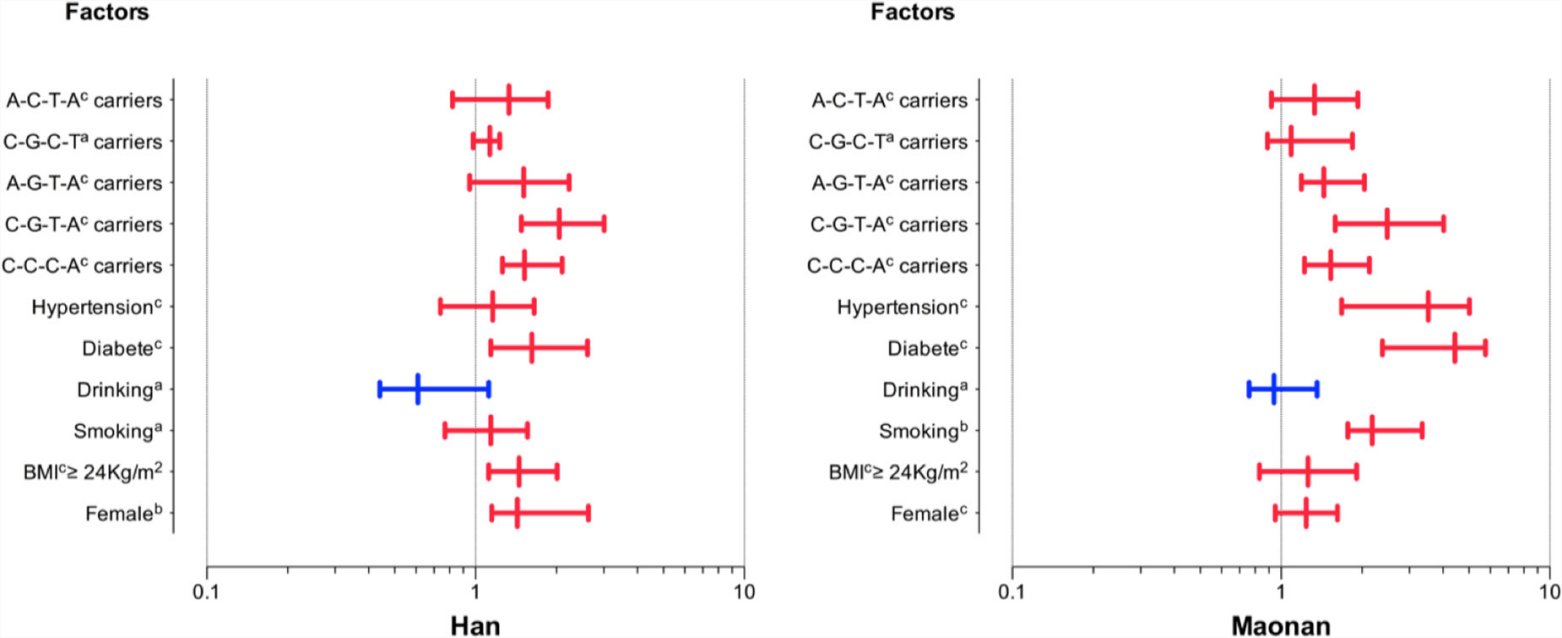
The stratified risk factors and *MVK/MMAB* haplotypes associated with dyslipidemia in the Han and Maonan populations Stratified analysis showed an increased risk of dyslipidemia in female, high BMI, smoking, diabetes or hypertension subgroups and haplotype carriers, but not in drinking subgroup. ^a^*P* < 0.05;^b^*P* < 0.01 and ^c^*P* < 0.001.

To detect the integrated effect of four SNPs (the combined sequence was rs3759387, rs877710, rs7134594 and rs9593) in the cluster, a linkage disequilibrium (LD) was noted between four SNPs (Supplementary Figure 1). Five haplotypes were identified in the cluster in both populations, and the rare haplotypes (frequency < 3%) have been dropped. The haplotype of rs3759387C-rs877710C-rs7134594C-rs9593T was the commonest one (57.23%). All of the haplotypes had a significant meaning (*P* < 0.001). The haplotypes associated with the risk of dyslipidemia in both ethnic groups are also shown in Table 2. The effects of these haplotypes on serum lipid traits are shown in Figure 3. In the meantime, the stratified risk factors and *MVK/MMAB* haplotypes associated with dyslipidemia in each of (Han or Maonan) populations are clarified in Figure 4.

**Table 2:** Haplotype frequencies among the 4 SNPs in the Maonan and Han populations [n (%)], and associated with dyslipidemia in both ethic groups

		Haplotype		Total	Han	Maonan	*P*-value	OR (95%CI)
A	B	C	D					
C	C	C	T	1439(57.23)	721(57.65)	718(56.82)	0.000111	0.80(0.71∼0.89)
C	G	T	A	367(14.60)	126(10.06)	241(19.02)	5.55×10^-16^	1.99(1.69∼2.35)
A	G	T	A	312(12.41)	141(11.27)	171(13.55)	0.007179	1.17(0.97∼1.34)
C	G	C	T	199(7.91)	88(7.06)	111(8.78)	0.007975	1.20(0.98∼1.48)
A	C	T	A	49(1.93)	36(2.86)	13(0.53)	1.52×10^-16^	0.16(0.09∼0.32)

A, rs3759387 A > C; B, rs877710 C > G; C, rs7134594 C > T; D, rs9593 A > T.

### Correlated factors for serum lipid parameters

Table 3 descripts the association between serum lipid profiles and four *MVK/MMAB* alleles and/or genotypes in the Han and Maonan ethnic groups, adjusting for age, sex, BMI, smoking status, alcohol use, and exercise. The genotypes and alleles were associated with HDL-C (rs3759387, rs877710 and rs7134594) in Maonan minority (*P* < 0.0125); HDL-C (rs3759387 and rs7134594) in Han nationality (*P* < 0.0125). In addition, serum lipid profiles were also related to some environmental exposures such as gender, time to life, alcohol drinking, cigarette smoking, blood pressure, blood glucose, waist circumference, and BMI in both ethnic groups or in males and females (*P* < 0.0125-0.001; Table 4).

**Table 3:** Association between serum lipid parameters and the *MVK/MMAB* alleles/genotypes in the Han and Maonan populations

Lipid	SNP	Affected allele/ Other allele	Affected genotype/ Other genotype	Std.error	Beta	*t*	*P*
Han plus Maonan							
TC	rs877710		GG+CG/CC	0.027	0.119	4.581	1.2×10^-5^
HDL-C	rs3759387		AC+CC/AA	0.004	0.169	4.943	1.4×10^-5^
	rs3759387	C/A		0.023	0.123	3.948	8×10^-4^
	rs877710		GG+CG/CC	0.026	0.237	4.351	9.3×10^-4^
	rs877710	G/C		0.031	0.146	4.332	9×10^-4^
	rs7134594		CT+TT/CC	0.056	0.478	2.237	0.006
	rs7134594	T/C		0.023	0.141	3.422	5×10^-4^
	rs9593		AT+TT/AA	0.015	0.216	2.241	0.005
	rs9593	T/A		0.044	0.147	3.958	6×10^-4^
ApoA1	rs7134594		CT+TT/CC	0.091	0.091	3.443	0.001
	rs7134594	T/C		0.013	-0.067	-3.048	0.002
Han							
HDL-C	rs3759387		AC+CC/AA	0.028	-0.081	-2.452	0.011
	rs3759387	C/A		0.054	0.054	2.818	0.004
	rs7134594		CT+TT/CC	0.028	0.211	2.226	0.008
	rs7134594	T/C		0.021	0.229	3.444	8.4×10^-4^
ApoA1	rs877710	G/C		0.025	0.136	4.352	9.6×10^-4^
Maonan							
HDL-C	rs3759387		AC+CC/AA	0.003	0.139	4.903	1.33×10^-5^
	rs3759387	C/A		0.033	0.111	3.978	7.13×10^-4^
	rs877710		GG+CG/CC	0.025	0.224	3.998	6.6×10^-4^
	rs877710	G/C		0.014	0.009	2.981	0.001
	rs7134594		CT+TT/CC	0.043	0.332	2.544	0.003
	rs7134594	T/C		0.023	0.147	3.258	7.3×10^-4^
ApoA1	rs877710	G/C		0.029	0.220	4.137	9.2×10^-4^

*TC*, total cholesterol; *HDL-C*, high-density lipoprotein cholesterol; Apo, apolipoprotein; *Beta*, standardized coefficient.

**Table 4:** Association between serum lipid parameters and relative factors in the Han and Maonan populations

Lipid	Risk factor	B	Std.error	Beta	*T*	*P*
Han plus Maonan						
TC	Waist circumference	0.017	0.005	0.127	3.303	0.001
	Age	0.009	0.002	0.113	3.958	9.45×10^-4^
	Alcohol consumption	-0.497	0.053	-0.222	-9.323	1.87×10^-6^
	Body mass index	0.007	0.002	0.070	2.881	0.005
TG	Waist circumference	0.169	0.038	0.106	4.500	4.34×10^-5^
	Age	0.388	0.126	0.089	3.068	0.002
	Body mass index	0.079	0.025	0.076	3.128	0.002
	Glucose	0.011	0.004	0.071	2.913	0.005
HDL-C	Waist circumference	-0.010	0.002	-0.206	-5.289	5.33×10^-5^
	Gender	0.148	0.034	0.167	4.403	3.29×10^-4^
	Cigarette smoking	0.085	0.032	0.083	2.655	0.008
	Alcohol consumption	0.098	0.032	0.107	3.027	0.003
LDL-C	Ethnic group	0.022	0.006	0.144	3.917	0.003
	Age	-0.013	0.005	-0.108	-2.556	0.011
	Alcohol consumption	0.141	0.042	0.152	2.718	0.007
	Gender	-0.007	0.003	-0.120	-2.040	0.012
ApoA1	Age	0.166	0.051	0.145	3.241	0.001
	Glucose	0.081	0.027	0.165	2.988	0.003
	Gender	0.163	0.027	0.268	6.067	7.13×10^-5^
	Ethnic group	0.080	0.026	0.146	3.152	0.002
ApoB	Glucose	0.009	0.004	0.085	2.466	0.005
	Ethnic group	0.028	0.010	0.147	2.731	0.006
ApoA1/ApoB	Ethnic group	0.540	0.170	0.127	3.177	0.002
	Age	-0.103	0.035	-0.479	-2.936	0.003
	Gender	-0.299	0.105	-0.620	-2.854	0.004
	Alcohol consumption	-0.012	0.002	-0.273	-5.069	4.41×10^-5^
Han						
TC	Waist circumference	0.021	0.003	0.336	6.305	7.88×10^-5^
	Age	-0.034	0.012	-0.904	-2.938	0.005
TG	Waist circumference	-0.127	0.040	-1.691	-3.189	9.73×10^-4^
	Glucose	0.510	0.180	1.634	2.867	0.004
	Systolic blood pressure	0.004	0.001	0.218	4.646	4.03×10^-4^
HDL-C	Gender	-0.008	0.002	-0.203	-3.752	9.93×10^-4^
	Cigarette smoking	0.173	0.046	0.204	3.745	9.96×10^-4^
	Weight	0.297	0.073	0.216	4.409	5.92×10^-4^
	Alcohol consumption	0.079	0.030	0.144	2.614	0.009
LDL-C	Waist circumference	0.003	0.001	0.199	3.193	0.002
	Gender	-0.299	0.105	-0.620	-2.854	0.004
	Age	0.067	0.017	0.207	4.028	6.16×10^-4^
	Cigarette smoking	0.003	0.001	0.186	3.650	9.98×10^-4^
ApoA1	Gender	0.044	0.013	0.142	3.342	0.001
	Alcohol consumption	0.001	0.001	0.118	2.517	0.012
ApoB	Glucose	-0.003	0.001	-0.138	-2.922	0.004
	Age	0.001	0.001	0.079	2.034	0.011
ApoA1/ApoB	Glucose	-0.021	0.007	-0.091	-3.156	0.002
	Gender	0.147	0.019	0.173	3.805	9.97×10^-4^
Maonan						
TC	Waist circumference	0.316	0.141	1.131	2.549	0.010
	Gender	0.021	0.003	0.336	6.305	7.44×10^-5^
	Weight	0.297	0.073	0.216	4.409	5.22×10^-4^
	Age	-0.034	0.012	-0.904	-2.938	0.005
TG	Waist circumference	-0.127	0.040	-1.691	-3.189	9.98×10^-4^
	Alcohol consumption	0.510	0.180	1.634	2.867	0.004
	Height	0.012	0.003	0.150	3.653	9.95×10^-4^
	Weight	0.366	0.059	0.207	6.148	7.11×10^-5^
	Body mass index	0.028	0.010	0.147	2.731	0.006
HDL-C	Waist circumference	0.540	0.170	0.127	3.177	0.002
	Systolic blood pressure	-0.103	0.035	-0.479	-2.936	0.003
	Diastolic blood pressure	0.156	0.048	0.959	3.237	0.001
	Alcohol consumption	-0.012	0.002	-0.273	-5.069	5.23×10^-5^
	Cigarette smoking	0.157	0.044	0.192	3.601	9.92×10^-4^
LDL-C	Age	0.019	0.007	0.152	2.611	0.009
	Waist circumference	0.010	0.003	0.117	3.142	0.002
ApoA1	Alcohol consumption	0.061	0.013	0.273	4.743	0.006
	Cigarette smoking	0.022	0.006	0.144	3.917	0.003
ApoB	Waist circumference	-0.013	0.005	-0.108	-2.556	0.011
ApoA1/ApoB	Age	-0.007	0.003	-0.120	-2.940	0.004
	Alcohol consumption	0.166	0.051	0.145	3.241	0.001

*TC*, total cholesterol; *TG*, triglyceride; *HDL-C*, high-density lipoprotein cholesterol; *LDL-C*, low-density lipoprotein cholesterol; *ApoA1*, apolipoprotein A1; *ApoB*, apolipoprotein B; *ApoA1/ApoB*, the ratio of apolipoprotein A1 to apolipoprotein B; *B*, unstandardized coefficient; *Beta*, standardized coefficient.

### Relative factors for serum lipid phenotypes

**Figure 5:**
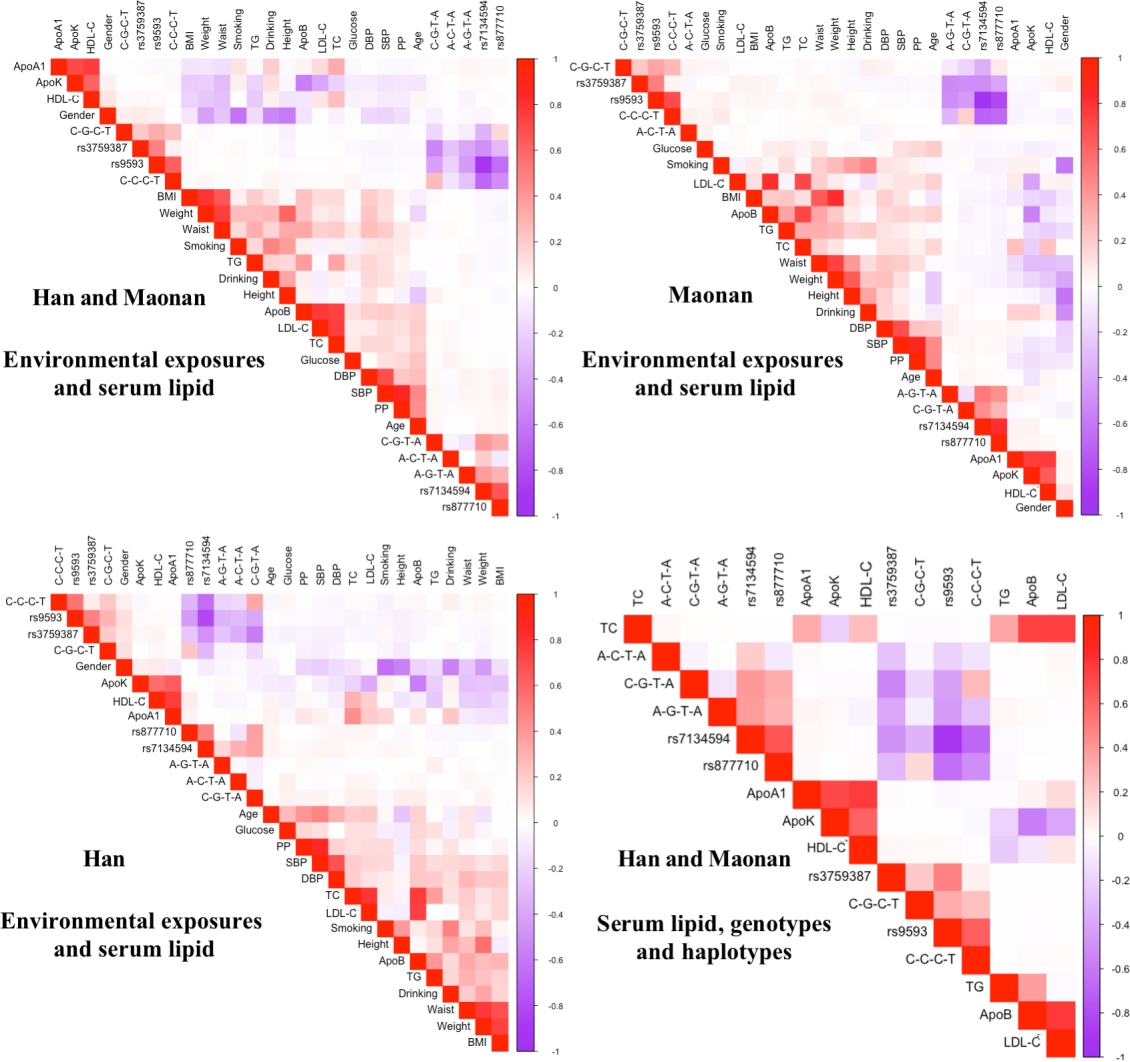
Correlation between environmental exposures and serum lipid variables, as well as the candidate loci *TC,* total cholesterol; *TG*, triglyceride; *HDL-C*, high-density lipoprotein cholesterol; *LDL-C*, low-density lipoprotein cholesterol; *ApoA1*, apolipoprotein A1; *ApoB*, apolipoprotein B; *ApoA1/ApoB*, the ratio of apolipoprotein A1 to apolipoprotein B; *BMI*, body mass index.

As shown in Figure 5, Pearson correlation analysis demonstrated that the integrative variants and haplotypes connected with the *MVK* rs3759387 and *MMAB* rs877710, rs7134594, rs9593 SNPs to lipid variables. A number of environmental exposures such as time to life, sex, cigarette smoking, alcohol drinking and conventional cardiovascular risk factors such as BMI and blood pressure values were also related to plasm lipid phenotypic profiles of both ethnic groups.

## DISCUSSION

In the current study, it is the first time we revealed the association of the *MVK* rs3759387 and *MMAB* rs877710, rs7134594, rs9593 SNPs and some serum lipid profiles in Maonan minority; the LD status, the haplotype frequencies of the selected SNPs and associated with dyslipidemia in Maonan and/or Han ethnic groups. Furthermore, we also completely duplicated the association of *MVK* rs3759387 and *MMAB* rs877710, rs7134594, rs9593 SNPs with the levels of serum HDL-C in the Maonan population; and *MVK* rs3759387 and *MMAB* rs877710, rs7134594, rs9593 SNPs with serum HDL-C levels and *MMAB* rs877710, rs7134594 SNPs with serum ApoA1 in the Han ethnic group. The SNPs of rs877710, rs3759387 and rs7134594 have been associated with HDL-C in some previous studies [24, 25], however nothing can be found about the association of the four SNPs and other serum lipid profiles in previous researches.

Besides, the differences in genotypic and allelic frequencies of four SNPs in current research also have not been found in different racial/ethnic groups previously. In the current study, we have shown that the genotypic and allelic frequencies of the four SNPs were distinguished Maonan from Han ethnic groups. All of the selected SNPs were in the Hardy-Weinberg equilibrium. According to the International 1000 Genomes data-base (https://www.ncbi.nlm.nih.gov/variation/tools/1000genomes/), the rs3759387 frequencies of A allele and AA, AG genotypes were 23.5%, 5.3% and 36.5% in European; the rs7134594 frequencies of C allele and CC, CT genotypes were 48.7%, 25.7% and 46.1% in European; the rs877710 frequencies of C allele and CC, CG genotypes were 48.7%, 25.6% and 46.0% in European; the rs9593 frequencies of A allele and AA, AT genotypes were 50%, 21.7% and 56.7% in European; respectively. All of above frequencies in European were significantly different from Han or Maonan ethnic groups. That means the minor allele or rare homozygote genotype frequencies in European ancestries of the four selected SNPs were different from Asian ethnic groups from the data. These outcomes reveal that the prevalence of the four SNPs’ minor allelic or rare homozygote genotypic frequencies would be shared a racial/ethnic-specificity.

*MVK* and *MMAB* are arranged in a head-to-head orientation on chromosome 12. In humans, when MVK mutations in homozygosity can give rise to hyperimmunoglobulinemia D syndrome, which the basic symptoms were fever and high concentrations of immunoglobulins D and A in blood. When the patients suffered from hyperimmunoglobulinemia D syndrome, low HDL-C levels can be found, in accordance with the latest GWASes findings [18, 19]. However, when somebody lacked of cob (I) alamin adenosyltransferase, as an enzyme encoded by MMAB, may contribute to methylmalonic aciduria [32]. But, the exact metabolism by which MMAB influences cholesterol is still unknown. A relevant report about schizophrenia had revealed that urinary methylmalonic acid may negatively correlated with red blood cell membrane cholesterol levels in blood [33]. In addition, *MVK* and *MMAB*, which share the same promoter, are both modified by sterol-responsive element-binding protein 2 (SREBP2), which is a transcription factor that controls cholesterol homeostasis. Furthermore, the way in which these two neighboring genes take part in metabolic pathways may have an effect on HDL metabolism had been found. *MVK* encodes for MVK, play an important role in an initial stage in cholesterol biosynthesis (Supplementary Figure 5) [34]. In contrast, when lacked of cob (I) alamin adenosyltransferase, as an enzyme encoded by MMAB, someone may result to methylmalonic aciduria. The exact reason *MMAB* can adjust cholesterol metabolism has not been identified, maybe cholesterol synthesis through *SREBP2* can explain our findings [35]. The precise mechanism of *MVK* and *MMAB* on serum lipid metabolism remains to be investigated, which may provide a promising target for medical therapy.

In the present research, we also showed that there might be a racial/ethnic specific association of the four SNPs and lipid parameters. The association of the other SNPs near *MVK/MMAB* and lipid profiles has been reported previously [36, 37]. However, no significant association between *MVK/MMAB* SNPs and HDL-C concentrations was selected in the Japanese population [38]. At the same time, Junyent *et al.* showed that the *MMAB* 3U3527G > C variants may result in the variation in HDL-C levels, particularly in those individuals with high carbohydrate consumptions [27]. With these situations, it’s still not exactly to comprehend the reason why these contradictions related to the selected SNPs and serum lipid profiles between both of these ethnic groups. This might be attributed to the distinctions in genetic factor to some extent. In addition, the interaction of gene-gene, gene-environment and environment-environment on lipid parameters remain to be interpreted. Previous researches have revealed that environmental exposures were significantly correlated with serum lipid profiles, including hypertension, obesity, physical activity, dietary patterns and lifestyle [39–45]. There was association of gender, age, BMI, cigarette smoking, alcohol consumption, blood pressure and serum lipid levels in both Maonan and Han populations. Above of the explored data demonstrated several environmental exposures may play dominant part in influencing lipid profiles. The dietary habits were different between the Han and Maonan populations. Rice is the Maonan people’s staple food supplemented with corn, sweet potato and other grains. Maonan people preferred to eat spicy and acid food with lots of oil and salt. This preference of high in carbohydrates may be related to the higher blood glucose levels, weight, BMI and waist circumference in Maonan than in Han people. As well as rich oil and salt can give rise to higher blood pressure, serum TC, LDL-C and ApoB levels in Maonan than in Han people. Previous studies proved that diet alone could account for the variability on serum lipid levels [45, 46]. In the current study, we found that part of participants who consumed alcohol (> 25 g/day) was lower in Maonan than in Han ethnic groups. Whether the alcohol consumption take effects on lipid profiles was still not understood, maybe some differences would exist in kinds of specific patient or the alcohol consumption types, and it seems by different gender and ethnic groups. Thus, we just had better pay more attention to current studies [47].

Another reason might be attributed to the differences in LD pattern among the study ethnic groups. In the present study, we selected that the C-G-T-A, A-G-T-A and A-C-T-A haplotypes’ frequencies were distinguished Maonan from Han ethnic groups. The haplotypes which combined with four SNPs had more power to account for serum lipid variation than any single SNP alone, particularly to HDL-C. For this reason, ethnic distinctions in the LD pattern may account for the contradictions related to the selected SNPs with lipid profiles among diverse ethnic groups.

Besides above, the environmental exposures, genetic factors might also give rise to dyslipidemia [48]. When considered family and twin studies, the genetic factors can explain approximate 40%-60% of the person variation in serum lipid levels [49–51]. Intra-ethnic marriages were popular in Maonan. For example, more than 80% of the Maonan people share the same surname: Tan. Thus, the hereditary characteristics and phenotypes of some lipid metabolism-related genes in Maonan might be different from those in Han. A lot of series reports have found that the genetic polymorphisms of some genes in the Maonan population were different from those in Han Chinese [30, 52]. But this remains to be conclusively determined.

A few potential limitations cannot be ignored. First, compared to many GWASes and replication studies, our sample numbers were relatively small. With these situations, larger sample numbers are needed to determine the consequences in future studies. Next, lots of items cannot be matched in both ethnic groups, including the percentages of smoking or drinking, waist circumference and the body weight. In addition, several confounding factors may have potential effect on serum lipid levels among different genotypes in both ethnic groups, just as time to life, BMI, blood pressure, cigarette smoking, and alcohol consumption, even if we have been adjusted for the statistical analysis. Last but not least, because of not cover the extensive *MVK/MMAB* locus, a lot of messages from other SNPs would be drop out.

In summary, the genotypic and allelic frequencies of the *MVK* rs3759387 and *MMAB* rs877710, rs7134594 and rs9593 SNPs were different between Maonan and Han. Four SNPs were associated with HDL-C in the both ethnic groups; and one SNP with ApoA1 (rs7134594) in Han Chinese. There were five haplotypes identified in our study population. The *MVK* and *MMAB* SNPs and C-C-C-T, C-G-T-A, A-G-T-A, A-C-T-A haplotypes are associated with serum lipid traits. The haplotypes had more power to account for serum lipid variation than any single SNP alone, particularly to HDL-C.

## MATERIALS AND METHODS

### Subjects

The study populations including 1251 unrelated subjects (476 males, 38.05% and 775 females, 61.95%) of Han and 1264 unrelated participants (517 males, 40.83% and 747 females, 59.17%) of Maonan were randomly selected from our previous stratified randomized samples. The participants were all agricultural workers from Huanjiang Maonan Autonomous County, Guangxi Zhuang Autonomous region, People’s Republic of China. The participants’ age ranged from 25 to 80 years with the mean age of 55.88 ± 13.89 years in Han and 56.97 ± 15.12 years in Maonan; respectively. The age distribution and gender ratio were matched between the two groups. All participants were essentially healthy with no history of cardiovascular disease such as CAD, stroke, diabetes, hyper- or hypo-thyroids, and chronic renal disease. They were free from medications known to affect serum lipid levels. The investigations were carried out following the rules of the Declaration of Helsinki of 1975 (http://www.wma.net/en/30publications/10policies/b3/), revised in 2008. The study design was approved by the Ethics Committee of the First Affiliated Hospital, Guangxi Medical University (No: Lunshen-2014-KY-Guoji-001; Mar. 7, 2014). All procedures were performed in accordance with ethical standards. Informed consent was obtained from all participants.

### Epidemiological survey

The epidemiological survey was carried out using internationally standardized methods, following a common protocol [53]. Information on demographics, socioeconomic status, and lifestyle factors was collected with standardized questionnaires. Alcohol consumption was categorized into groups of grams of alcohol per day: 0 (non-drinker), < 25 and ≥ 25. Smoking status was categorized into groups of cigarettes per day: 0 (non-smoker), < 20 and ≥ 20. Several parameters such as blood pressure, height, weight, waist circumference, and BMI were measured. The methods of measuring above parameters were referred to a previous study [54].

### Biochemical analysis

A fasting venous blood sample of 5 ml was drawn from the participants. A part of the sample (2 mL) was collected into glass tubes and used to determine serum lipid levels. Another part of the sample (3 mL) was transferred to tubes with anticoagulants (4.80 g/L citric acid, 14.70 g/L glucose and 13.20 g/L tri-sodium citrate) and used to extract deoxyribonucleic acid (DNA). Measurements of serum TC, TG, HDL-C, and LDL-C levels in the samples were performed by enzymatic methods with commercially available kits (RANDOX Laboratories Ltd., Ardmore, Diamond Road, Crumlin Co. Antrim, United Kingdom, BT29 4QY; Daiichi Pure Chemicals Co., Ltd., Tokyo, Japan). Serum ApoA1 and ApoB levels were detected by the immunoturbidimetric immunoassay using a commercial kit (RANDOX Laboratories Ltd.). All determinations were performed with an auto-analyzer (Type 7170A; Hitachi Ltd., Tokyo, Japan) in the Clinical Science Experiment Center of the First Affiliated Hospital, Guangxi Medical University [55, 56].

### SNPs selection

We selected four SNPs in the *MVK/MMAB* with the following steps: (1) *MVK* gene clusters, which were selected from previous GWAS associated with lipid-metabolism. *MMAB* gene clusters are found to be close to *MVK* gene clusters and associated with serum lipid level especially HDL. (2) Tagging SNPs, which were established by Haploview (Broad Institute of MIT and Harvard, USA, version 4.2) and functional SNPs predicted to lead to serum lipid changes from current version of online resource (1000 Genome Project Database). (3) SNPs information was obtained from NCBI dbSNP Build 132 (http://www.ncbi.nlm.nih.gov/SNP/); (4) SNPs were restricted to minor allele frequency (MAF) > 1%; (5) SNPs might be associated with the plasma lipid levels or cardiovascular disease in recent studies; and (6) *MVK* rs3759587, rs7134594 and *MMAB* rs9593 rs877710, which were selected by the block-based approach. This strategy is enable by the correlations between tagging SNPs as manifested as LD. Although classic is not goal of tagging SNP selection, innovative tagging SNPs selection bias is inevitable.

### DNA amplification and genotyping

Genomic DNA of the samples was isolated from peripheral blood leucocytes according to the phenol-chloroform method [55, 56]. Genotyping of 4 mutations was performed by PCR-RFLP and Sanger sequencing. The characteristics of each mutation and the details of each primer pair, annealing temperature, length of the PCR products are summarized in Supplementary Table 1 and Supplementary Figures 2 and 3. The PCR products of the samples were sequenced with a sequencer ABI Prism 3100 Genetic Analyzer (Applied Biosystems, International Equipment Trading Ltd., Vernon Hills, IL, USA) in Shanghai Sangon Biological Engineering Technology & Services Co. Ltd., Shanghai China (Supplementary Figure 4).

### Diagnostic criteria

The normal values of serum TC, TG, HDL-C, LDL-C, ApoA1, ApoB levels and the ApoA1/ApoB ratio in our Clinical Science Experiment Center were 3.10–5.17, 0.56–1.70, 1.16–1.42, 2.70–3.10 mmol/L, 1.20–1.60, 0.80–1.05 g/L and 1.00–2.50, respectively. The individuals with TC > 5.17 mmol/L and/or TG > 1.70 mmol/L were defined as hyperlipidaemic [57]. Hypertension was diagnosed according to the 1999 and 2003 criteria of the World Health Organization-International Society of Hypertension Guidelines for the management of hypertension [58, 59]. The diagnostic criteria of overweight and obesity were according to the Cooperative Meta-analysis Group of China Obesity Task Force. Normal weight, overweight and obesity were defined as a BMI < 24, 24–28 and > 28 kg/m^2^, respectively [60].

### Statistical analyses

The statistical analyses were performed with the statistical software package SPSS 22.0 (SPSS Inc., Chicago, Illinois). The quantitative variables were presented as mean ± standard deviation (Because serum TG was not a normal distribution, the levels were presented as medians and interquartile ranges and analysis by Wilcoxon-Mann-Whitney test). Allele frequency was determined via direct counting, and the Hardy-Weinberg equilibrium was verified with the standard goodness-of-fit test. The genotype distribution between the two groups was analyzed by the chi-square test. General characteristics between two ethnic groups were compared by the Student’s unpaired *t*-test. The association between genotypes and serum lipid parameters was tested by covariance analysis (ANCOVA). Any SNPs associated with the lipid profiles at the value of *P* < 0.0125 (corresponding to *P* < 0.05 after adjusting for 4 independent tests by the Bonferroni correction) were considered statistically significant. Gender, age, BMI, blood pressure, alcohol consumption and cigarette smoking were adjusted for the statistical analysis. Haploview (Broad Institute of MIT and Harvard, USA, version 4.2) analyzed the haplotype frequencies and pair-wise LD among the detected SNPs. Unconditional logistic regression was used to assess the correlation between the risk of hyperlipidemia and genotypes. The model of age, gender, BMI, waist circumference, systolic blood pressure, diastolic blood pressure, pulse pressure, cigarette smoking, alcohol consumption and fasting plasma glucose level were adjusted for the statistical analysis. Multivariable linear regression analyses with stepwise modeling were used to determine the correlation between the genotypes (common homozygote genotype = 1, heterozygote genotype = 2, rare homozygote genotype = 3) or alleles (the minor allele non-carrier = 1, the minor allele carrier = 2) and several environmental factors with serum lipid levels in males and females of Han and Maonan populations. Two sides *P* value < 0.05 was considered statistically significant. The heart-map of inter-locus models was measured by R software (version 3.3.0) [61].

## SUPPLEMENTARY MATERIALS FIGURES AND TABLE


