# Supplementary material for: Association between the MVK and MMAB polymorphisms and serum lipid levels
Source: Oncotarget. 2017 Jul 31;8(41):70378–93. doi: 10.18632/oncotarget.19707 (PMC5642562; doi:10.18632/oncotarget.19707)
Supplement: Supplementary file 1 [file oncotarget-08-70378-s001.pdf]

Association between the *MVK* and *MMAB* polymorphisms and serum lipid levels

SUPPLEMENTARY MATERIALS

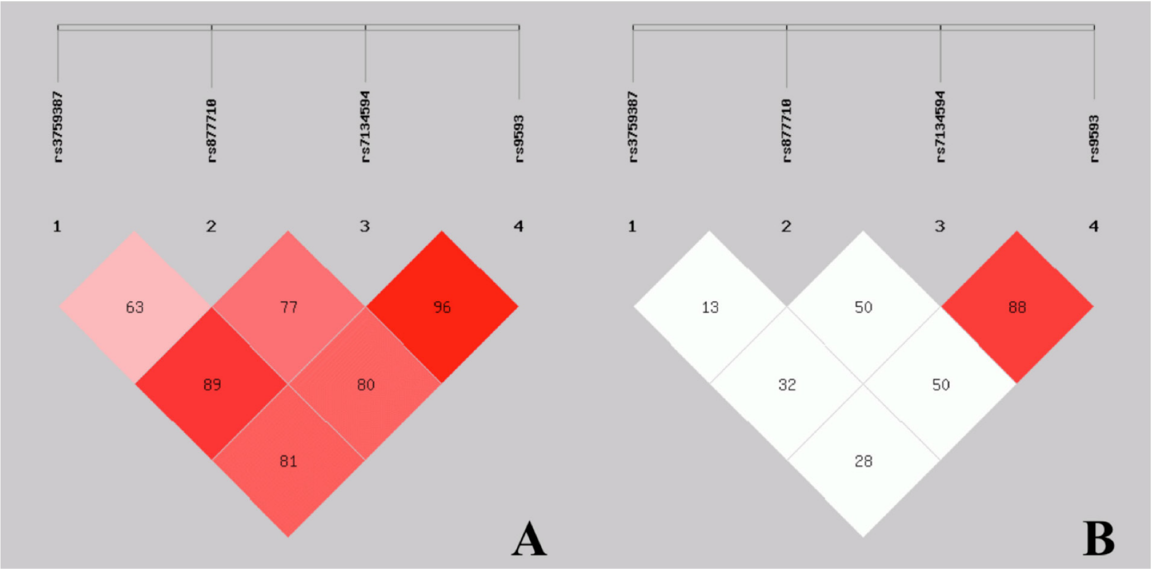

Supplementary Figure 1: **The linkage disequilibrium (LD) of the *MVK* rs3759387, *MMAB* rs877710, *MMAB* rs7134594 and *MMAB* rs9593 SNPs in the combined population of Maonan and Han. (A)** *D'*; **(B)** r^2.

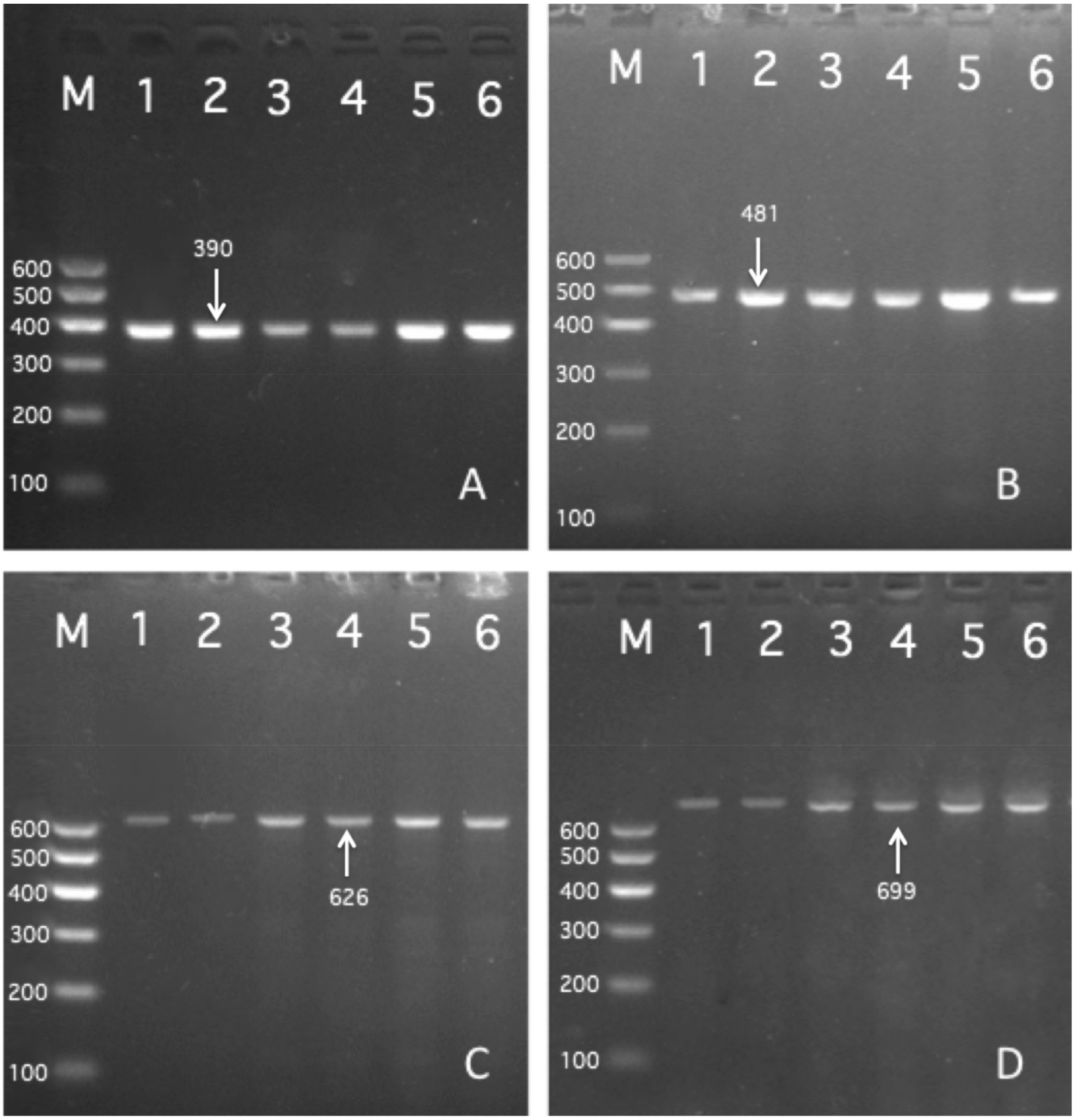

Supplementary Figure 2: Agarose gel electrophoresis (2%) of PCR products of the *MVK* and *MMAB* SNPs. Lane M, 100 bp DNA ladder; the PCR products of *MMAB* rs9593 **(A)**, *MMAB* rs7134594 **(B)**, *MVK* rs3759387 **(C)**, and *MMAB* rs877710 **(D)** SNPs were 390-, 481-, 626- and 699-bp nucleotide sequences; respectively.

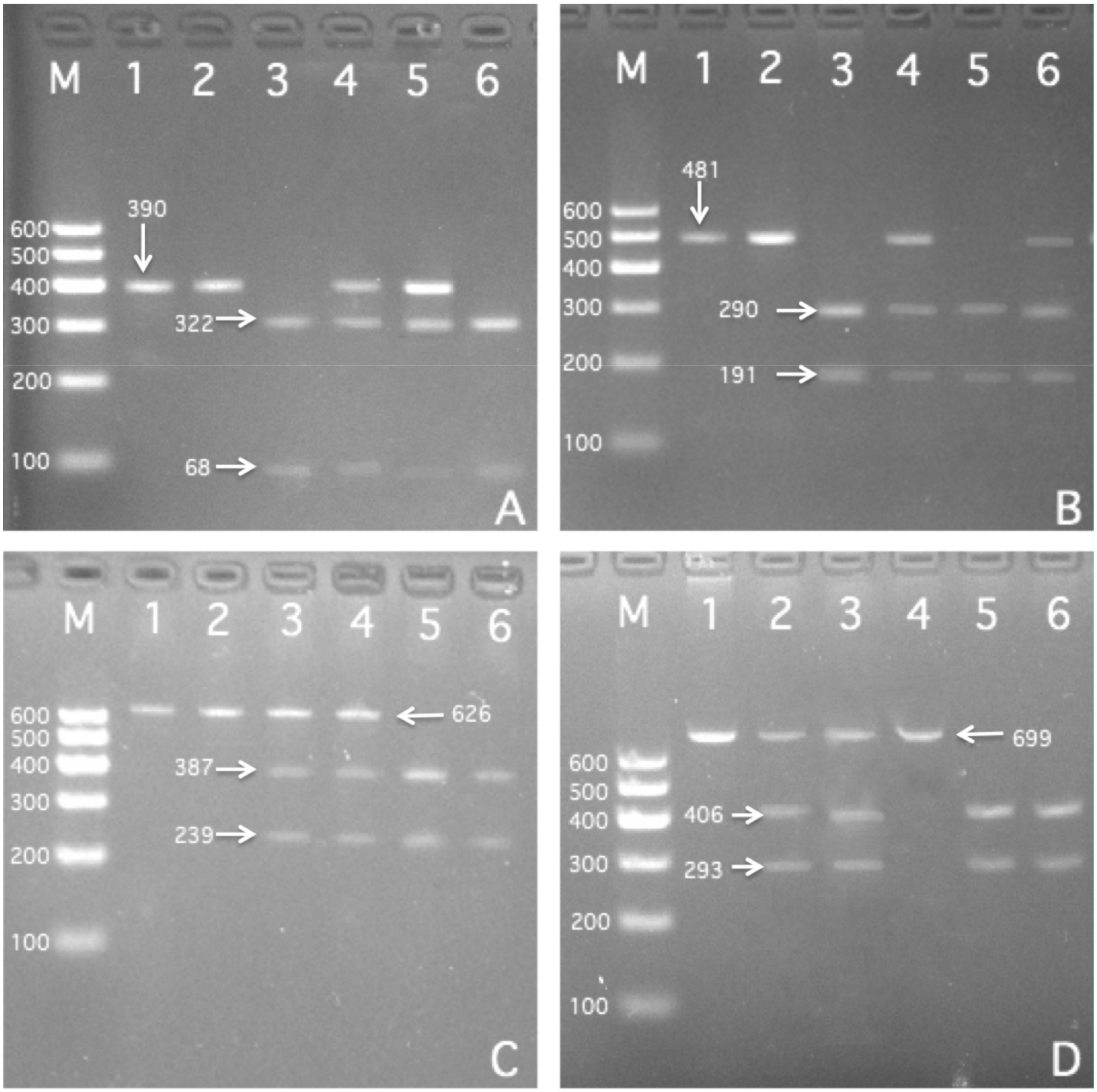

Supplementary Figure 3: Genotyping of the *MVK* and *MMAB* SNPs. Lane M, 100 bp DNA ladder. The genotypes of 4 SNPs were as follow: **(A)** *MMAB* rs9593: AA (lanes 1 and 2, 390-bp); AT (lanes 4 and 5, 390-, 322- and 68-bp); and TT genotype (lanes 3 and 6, 322- and 68-bp). **(B)** *MMAB* rs7134594: CC (lanes 1 and 2, 481-bp); CT (lanes 4 and 6, 481-, 290- and 191-bp); and TT genotype (lanes 3 and 5, 290- and 191-bp). **(C)** *MVK* rs3759387: AA (lanes 1 and 2, 626-bp); AC (lanes 3 and 4, 626-, 387-, 239-bp); and CC genotype (lanes 5 and 6, 387- and 239-bp). **(D)** *MMAB* rs877710: CC (lanes 1 and 4, 699- bp); CT (lanes 2 and 3, 699-, 406- and 293-bp); and TT genotype (lanes 5 and 6, 406- and 293-bp).

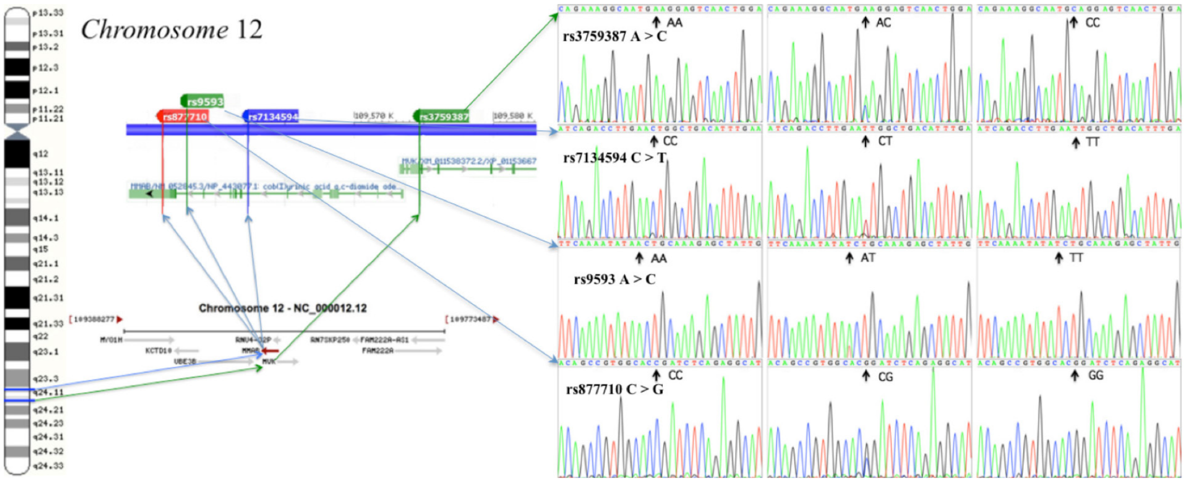

Supplementary Figure 4: The positions and parts of the nucleotide sequences of the *MVK* and *MMAB* SNPs. *MVK*, mevalonate kinase; *MMAB*, methylmalonic aciduria (cobalamin deficiency) cblB type.

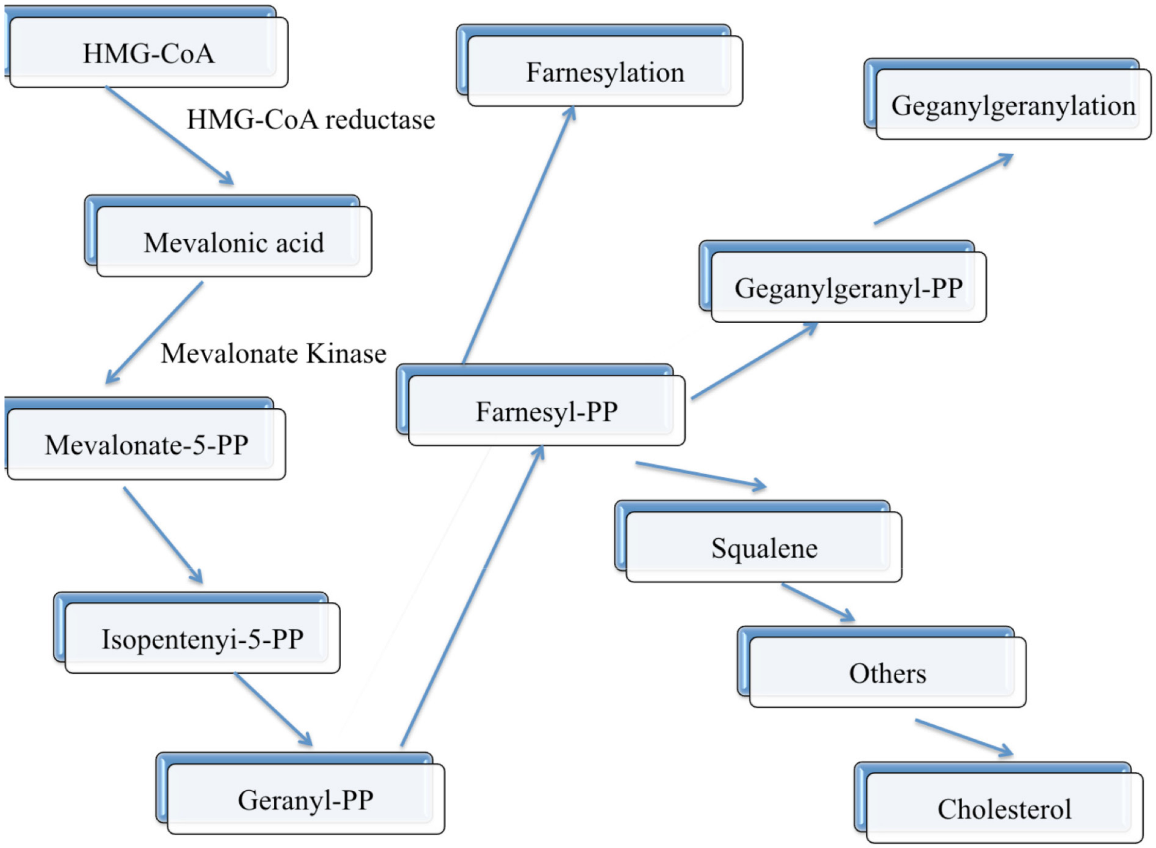

Supplementary Figure 5: Schematic representation of mevalonate pathway. The enzymes (HMG-CoA reductase and mevalonate kinase) are indicated along the pathway in bold characters.

Supplementary Table 1: The sequences of forward and backward primers of the *MVK-MMAB* mutations

SNV	Primer sequence	Annealing temperature	PCR product	Enzyme
MVK				
rs3759387	TACCACGGCATGTTCTCCAT	61°C	626bp	*HpyCH4*V
	GGCGTACTGGGTGTTTTCTG			
rs877710	AATATACCTGGCCCTCCTGC	61°C	699bp	*BstX2*I
	GGGGAAGGATAGCTGCAGAT			
MMAB				
rs9593	TCTCTCTGCCCCTCCAGACT	61°C	390bp	*Fae*I
	TGAGAAGGAGCTGTGCGGAA			
rs7134594	CCCCTAGTGCTGTCTTGTGA	61°C	481bp	*Bse1*I
	CCAGACGGTTCCTCTTGACT			
